# Reduced Life Expectancy due to Smoking in Large-Scale Cohort Studies in Japan

**DOI:** 10.2188/jea.JE2007416

**Published:** 2008-05-29

**Authors:** Kotaro Ozasa, Kota Katanoda, Akiko Tamakoshi, Hiroshi Sato, Kazuo Tajima, Takaichiro Suzuki, Shoichiro Tsugane, Tomotaka Sobue

**Affiliations:** 1 Epidemiology for Community Health and Medicine, Kyoto Prefectural University of Medicine; 2 Cancer Information Services and Surveillance Division, Center for Cancer Control and Information Services, National Cancer Center; 3 Division of Clinical Trials, National Center for Geriatrics and Gerontology; 4 Environmental Health Sciences, Tohoku University Graduate School of Medicine; 5 Aichi Cancer Center Research Institute; 6 Department of Cancer Control and Statistics, Osaka Medical Center for Cancer and Cardiovascular Diseases; 7 Epidemiology and Prevention Division, Research Center for Cancer Prevention and Screening, National Cancer Center

**Keywords:** Smoking, Life Expectancy, Cohort Studies

## Abstract

**Background:**

To show the reduction in life expectancy due to smoking and the recovery of normal life expectancy by smoking cessation is useful for tobacco control health policy.

**Methods:**

This study included 140,026 males and 156,810 females aged 40-79 years, who were participants of large-scale cohort studies in Japan (Japan Health Center-based Prospective Study [JPHC]-I, JPHC-II, Three-Prefecture Study, and Japan Collaborative Cohort [JACC] Study), which commenced around 1990. The mean follow-up period (±standard deviation) was 9.6 ± 2.3 years, during which 16,282 men and 9,418 women died. For persons aged 40-79 years grouped according to each defined smoking status in the baseline questionnaire, sex- and age-specific death rates at attained ages were calculated. The age-specific death rate was calculated by dividing the number of persons who died at the age by the number of persons who were followed-up at the attained age. From these death rates, current life tables were constructed according to the smoking status, and survival curves were plotted.

**Results:**

The life expectancy of male smokers, ex-smokers, and never-smokers at age 40 years was 38.5, 40.8, and 42.4 years respectively. In women, the corresponding life expectancies were 42.4, 42.1, and 46.1 years. In both sexes, the age by which half of the current smokers had died was approximately 4 years younger than that for never-smokers. The life expectancies of male ex-smokers who quit smoking before ages 40, 50, 60, and 70 years were 4.8, 3.7, 1.6, and 0.5 years longer than those of smokers, respectively.

**Conclusion:**

Smoking considerably reduced the life expectancy, and earlier smoking cessation resulted in a better survival than that seen with continued smoking.

## INTRODUCTION

Smoking is a major cause of cancers and cardiovascular and other diseases and consequently increases the risk of death.^1^ Determining the reduced life expectancy due to smoking in addition to the increased risk of developing individual diseases should be effective in estimating the health burden associated with smoking. Survival curves derived from life tables have revealed a trend toward increased mortality with age. Several studies have used survival curves to investigate the reduction in life expectancy caused by smoking.^2^^-^^9^ A previous Japanese study investigating circulatory diseases was based on a representative population in Japan but included a relatively small sample size.^10^

Several large-scale cohort studies based on the general population of Japan commenced in the early 1990s,^11^^-^^14^ and around 300,000 middle-aged and elderly men and women were observed over approximately 10 years. In the present study, we sought to construct life tables to evaluate the reduction in life expectancy due to smoking and to assess whether normal life expectancy is regained by the cessation of smoking at different ages.

## METHODS

The subjects of this study were derived from 3 cohort studies in Japan.^11^^-^^15^ One of these was the Three-Prefecture Study, which was conducted in Miyagi, Aichi, and Osaka Prefectures in a total of 15 areas. The study population comprised all residents aged 40 years or older in the study areas. The subjects were followed up from February 1, 1983/December 1, 1990 through December 31, 1993/February 28, 2000 (different follow-up periods in different study areas).^11^ The second study was the Japan Collaborative Cohort (JACC) Study, which was conducted in 45 areas in the 4 main islands of Japan, excluding Shikoku Island. The study population consisted of all residents aged 40 years or older in 22 areas, participants of health examinations conducted by the municipalities in 20 areas, and a combination of the above and atomic bomb survivors in the remaining areas. The subjects were followed up from 1988/1990 through December 31, 1999 (different starting periods in different areas).^12^^,^^13^ The third study was the Japan Public Health Center-based Prospective (JPHC) Study.^14^ The subjects of JPHC-I were recruited in 1990, and this study targeted registered inhabitants aged 40-59 years in 5 public health center areas in Iwate, Akita, Nagano, Okinawa, and Tokyo. They were followed up from January 1, 1990 to December 31, 2000.^15^ JPHC-II subjects were recruited in 1993-1994, and this study targeted registered inhabitants aged 40-69 years in 6 public health center areas in Niigata, Ibaraki, Kochi, Nagasaki, Okinawa, and Osaka. They were followed up from January 1, 1993 through December 31, 2003.^15^

Smoking status was surveyed in the baseline questionnaire for each cohort study. The status was divided into 3 categories: smoking, ex-smoking, and nonsmoking. Among smokers and ex-smokers, the age at which smoking was started (and stopped in the case of ex-smokers) and the number of cigarettes consumed a day were recorded.^11^^-^^15^ Follow up of the subjects was study specific, and the details are described elsewhere.^11^^-^^15^ As a general rule, all deceased people in each study area and people who moved out of the study area were identified using the population registry in each municipality office. The cause of death for each person was obtained from the death certificate.

A total of 140,026 men and 156,810 women aged 40-79 years from the 3 cohorts were included in this study (Table 1). The mean follow-up period (±standard deviation) was 8.5 ± 2.7 years, 9.9 ± 2.2 years, 10.4 ± 1.6 years, and 10.2 ± 1.7 years for the Three-Prefecture Study, JACC Study, and JPHC-I and -II Studies, respectively. The prevalence of smokers in each cohort is shown in Table 1. The age-adjusted death rate by cohort, sex, and smoking status was separately calculated for the age groups of 40-69 years and 70 years or older and based on the sex- and 5-year age-specific death rates was classified according to the smoking status of each cohort. The standard population was constituted of age-specific numbers of persons who were followed-up at the attained age. The adjusted death rates in smokers and nonsmokers varied slightly between cohorts, and the rate ratios of smokers/nonsmokers were approximately 1.5-1.8 in men and 1.4-2.1 in women.

**Table 1.** Characteristics of the cohorts.

	Cohort study	No.	Age at baseleine survey (years) (Mean±SD, ranege)	Prevalence of current smokers	Age-adjusted death rate at attained age groups ‡ (per 1,000)			

					40-69 years old		70 years or older	

					Smokers	Never-smoker	Smokers	Never-smoker
Male	Three-Prefecture	44,453	54.4 ± 10.2 (40-79)	57.8%	9.31	5.79	54.0	36.2
	JACC *	42,528	57.3 ± 10.2 (40-79)	53.0%	7.62	4.51	44.9	28.1
	JPHC †-I	23,478	49.0 ± 6.0 (40-59)	53.6%	8.24	5.28	NA	NA
	JPHC-II	29,567	53.2 ± 8.8 (40-69)	52.0%	9.32	4.96	41.4	24.1
	Total	140,026	54.1 ± 9.7 (40-79)	54.4%	8.59	5.01	48.2	30.9


Female	Three-Prefecture	43,704	55.2 ± 10.5 (40-79)	11.9%	6.21	3.62	33.8	25.0
	JACC	53,370	57.3 ± 10.1 (40-79)	5.6%	4.72	2.44	28.3	17.2
	JPHC-I	26,561	49.1 ± 5.9 (40-59)	7.9%	4.25	2.91	NA	NA
	JPHC-II	33,175	53.5 ± 8.9 (40-69)	7.3%	5.85	2.80	18.6	12.4
	Total	156,810	54.5 ± 9.8 (40-79)	8.1%	5.53	2.87	31.3	19.3

* JACC: Japan Collaborative Cohort

† JPHC: Japan Public Health Center-based prospective Study Cohort

‡ Adjusted for the standard population constituted of age-specific person years observed at the attained age.

NA: not available

The characteristics of all the subjects are shown in Table 2. The mean age (±standard deviation) was 54.1 ± 9.7 years in men and 54.5 ± 9.8 years in women. The prevalence of male smokers was 54.4% overall and 59.5%, 54.2%, 55.6%, and 42.5%, respectively, for the age groups of 40-49, 50-59, 60-69, and 70-79 years. There were 25.1% male ex-smokers and 20.5% male never-smokers. Amongst women, 8.1% were smokers (9.5%, 7.5%, 6.8%, and 8.5%, respectively, for the age groups mentioned above); 2.4% were ex-smokers; and 89.5% were never-smokers. The smokers were further classified according to the number of cigarettes consumed per day, and the ex-smokers were categorized by the age at which they stopped smoking.

**Table 2.** Characteristics at the baseline survey of subjects and observed person-years from all cohorts.

	No.	Observed person- years	Age at baseleine survey (years) (Mean ± SD)	No. of cigarettes consumed a day (Mean ± SD)	Duration of smoking (years) (Mean ± SD)	Age at quitting (years) (Mean ± SD)	Duration after quitting smoking (years) (Mean ± SD)
Male							
Smokers	76,227	717,200	53.2 ± 9.4	22.3 ± 10.9	32.2 ± 9.6	NA	NA
Ex-smokers	35,079	328,883	56.4 ± 10.0	23.3 ± 13.6	24.7 ± 11.9	45.4 ± 12.0	10.8 ± 8.9
Never-smokers	28,720	278,921	53.7 ± 9.5	NA	NA	NA	NA
Total	140,026	1,325,004	54.1 ± 9.7	NA	NA	NA	NA

(subgroups)							
Smokers, 1-14 cigarettes a day	12,838	117,742	56.8 ± 10.3	8.9 ± 2.6	34.5 ± 11.3	NA	NA
Smokers, 15-24 cigarettes a day	37,845	357,916	53.9 ± 9.4	18.9 ± 2.0	33.0 ± 9.6	NA	NA
Smokers, 25+ cigarettes a day	24,374	230,170	50.2 ± 7.9	34.7 ± 9.4	30.0 ± 8.1	NA	NA

Ex-smokers, quit at age <40 years	10,384	100,155	49.0 ± 8.3	21.6 ± 13.6	11.8 ± 5.2	31.7 ± 5.0	17.2 ± 9.8
Ex-smokers, quit at age 40-49 years	10,122	97,328	53.5 ± 7.7	24.8 ± 14.1	22.9 ± 4.4	43.6 ± 2.9	9.9 ± 7.8
Ex-smokers, quit at age 50-59 years	7,917	74,227	61.1 ± 5.7	24.1 ± 13.4	32.5 ± 5.1	53.6 ± 2.9	7.5 ± 5.8
Ex-smokers, quit at age 60-69 years	3,962	34,117	68.0 ± 4.4	22.7 ± 12.6	41.1 ± 5.6	62.8 ± 2.6	5.1 ± 4.3

Female							
Smokers	12,717	117,172	53.4 ± 10.1	14.3 ± 8.6	23.3 ± 11.4	NA	NA
Ex-smokers	3,714	33,517	56.5 ± 11.1	12.1 ± 9.0	17.6 ± 11.9	46.6 ± 13.0	9.6 ± 8.4
Never-smokers	140,379	1,379,703	54.5 ± 9.7	NA	NA	NA	NA
Total	156,810	1,530,392	54.5 ± 9.8	NA	NA	NA	NA

(subgroups)							
Smokers, 1-14 cigarettes a day	6,296	58,029	54.4 ± 10.7	7.9 ± 2.9	22.3 ± 12.4	NA	NA
Smokers, 15-24 cigarettes a day	4,944	45,497	52.4 ± 9.5	18.2 ± 2.3	24.1 ± 10.4	NA	NA
Smokers, 25+ cigarettes a day	1,061	9,570	50.7 ± 8.4	34.0 ± 8.9	25.4 ± 9.4	NA	NA

Ex-smokers, quit at age <40 years	956	8,923	46.2 ± 8.0	10.4 ± 7.8	8.1 ± 5.5	30.6 ± 5.5	15.5 ± 9.3
Ex-smokers, quit at age 40-49 years	879	8,027	52.7 ± 7.9	11.9 ± 9.1	15.4 ± 7.9	43.8 ± 3.0	8.9 ± 8.2
Ex-smokers, quit at age 50-59 years	869	7,966	60.7 ± 6.1	13.0 ± 9.0	20.6 ± 9.6	53.4 ± 3.0	7.3 ± 6.1
Ex-smokers, quit at age 60-69 years	469	3,966	68.2 ± 4.3	14.1 ± 10.3	29.2 ± 11.1	62.6 ± 2.6	5.5 ± 4.5

NA: not available, SD: standard deviation

Sex- and age-specific death rates were calculated based on the observed person-years at attaining ages and the number of deaths at that age. From these death rates, the complete current life tables were constructed using Chiang’s method, for each smoking status.^16^ Life expectancies at age 40 years were calculated, and survival curves beginning at age 40 years up to that at age 90 years, for a population of 100,000, were plotted. We estimated the 95% confidence interval (CI) for life expectancy by setting the age intervals less than 90 years as one year and the last interval as age 90 years or older.^16^

For the ex-smoker subgroup, the age group at which the subjects quit smoking was divided into 10-year intervals, i.e., 40-49 years, 50-59 years, and 60-69 years. For those who quit smoking at the age of 40-49 years, the death rates up to the age of 44 years were assumed to be equal to those of smokers, while those at ages 45 or older were considered to be equal to the death rates of ex-smokers. Death rates for those who quit smoking at the age 50-59 years or 60-69 years were derived in the same way.

## RESULTS

**Figure 1:**
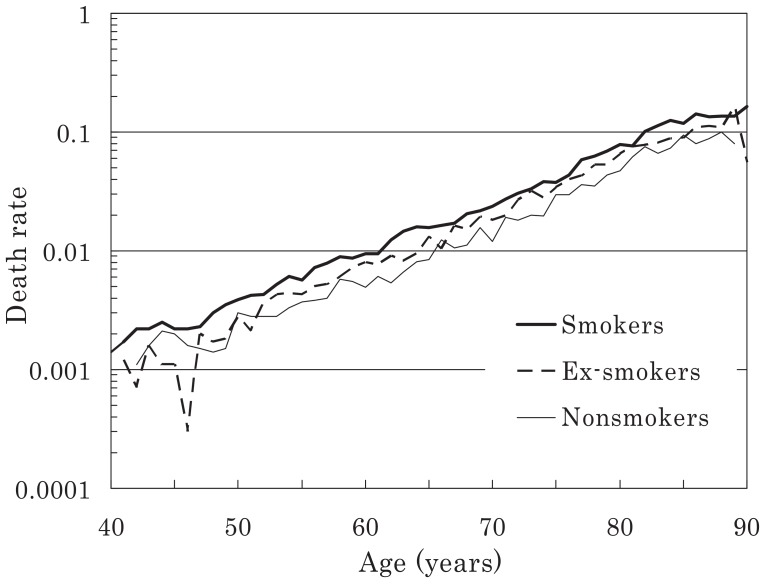
Age-specific death rates calculated at the attained ages (males).

**Figure 2:**
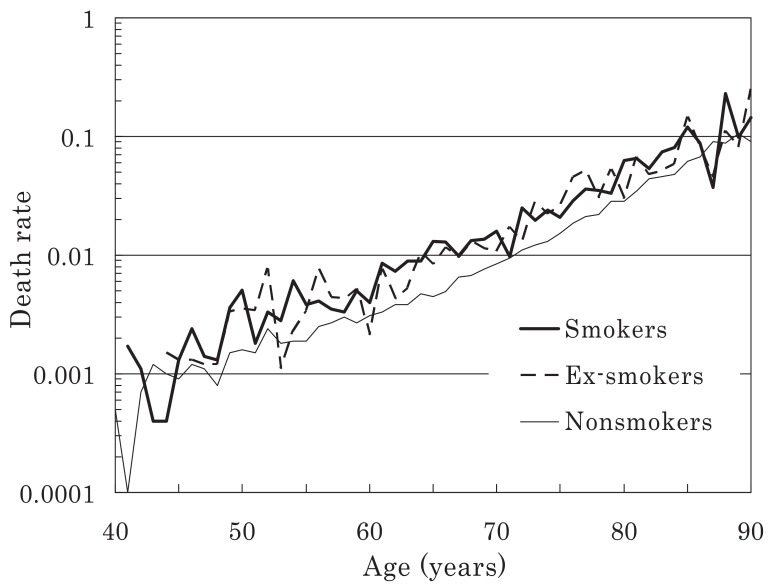
Age-specific death rates calculated at the attained ages (females).

The person-years by smoking status and the number of deaths among men and women are presented in Table 2. Most observations were distributed amongst subjects in their 50s and 60s. Sex- and age-specific death rates up to the age of 89 years are shown for men and women in Figures 1 and 2, respectively. In general, the rates increased in an exponential linear pattern in both sexes, regardless of the smoking status.

At age 40 years, the life expectancy was 38.5 years (95% CI: 38.3 and 38.7) for male smokers, 40.8 years (95% CI: 40.6 and 41.0) for ex-smokers, and 43.2 years (95% CI: 42.2 and 42.7) for never-smokers (Table 3). For women, the corresponding life expectancies at age 40 years were 42.4 (95% CI: 42.1 and 43.0), 43.1 (95% CI: 42.1 and 43.5), and 46.8 (95% CI: 46.0 and 46.3) years (Table 3). Both male and female heavy smokers had slightly shorter life expectancies than those of light smokers. Male ex-smokers who quit before age 40 years had a slightly longer life expectancy (43.3 years, 95% CI: 42.6 and 43.9) than that of never-smokers. Male ex-smokers who quit smoking at younger age had a longer life expectancy than that of ex-smokers who quit at older age.

**Table 3.** Observations on follow up, and calculated survival rates and life expectancies.

	Person-years of follow up	No. of deaths	Age by which half of the population died from age of 40 years	Life expectancy at age of 40 years (95% confidence interval)
Male				
Smokers	717,200	9,240	80.4	38.5 (38.3, 38.7)
Ex-smokers	328,883	4,582	82.4	40.8 (40.6, 41.0)
Never-smokers	278,921	2,460	84.6	42.4 (42.2, 42.7)
Total	1,325,004	16,282	81.8	39.9 (39.8, 40.0)

(subgroups)				
Smokers, 1-14 cigarettes a day	117,742	2,224	80.1	38.3 (37.8, 38.7)
Smokers, 15-24 cigarettes a day	357,916	4,762	80.6	38.7 (38.4, 38.9)
Smokers, 25+ cigarettes a day	230,170	2,111	79.8	37.9 (37.4, 38.4)

Ex-smokers, quit at age <40 years	100,155	474	86.1	43.3 (42.6 43.9)
Ex-smokers, quit at age 40-49 years	97,328	739	84.9	42.2 (41.7, 42.7)
Ex-smokers, quit at age 50-59 years	74,227	1,300	82.4	40.1 (39.6, 40.6)
Ex-smokers, quit at age 60-69 years	34,117	1,201	81.1	39.0 (38.3, 39.6)

Female				
Smokers	117,172	1,085	84.4	42.5 (42.1, 43.0)
Ex-smokers	33,517	409	85.0	42.8 (42.1, 43.5)
Never-smokers	1,379,703	7,924	88.4	46.1 (46.0, 46.3)
Total	1,530,392	9,418	88.0	45.7 (45.6, 45.9)

(subgroups)				
Smokers, 1-14 cigarettes a day	58,029	609	84.5	42.5 (41.9, 43.2)
Smokers, 15-24 cigarettes a day	45,497	366	84.0	42.3 (41.5, 43.0)
Smokers, 25+ cigarettes a day	9,570	79	81.9	40.4 (38.3, 42.4)

Ex-smokers, quit at age <40 years	8,923	28	85.0	43.5 (41.2, 45.9)
Ex-smokers, quit at age 40-49 years	8,027	52	89.0	43.9 (41.7, 46.0)
Ex-smokers, quit at age 50-59 years	7,966	94	86.5	43.9 (42.6, 45.3)
Ex-smokers, quit at age 60-69 years	3,966	106	84.9	42.0 (41.0, 43.0)

**Figure 3.**
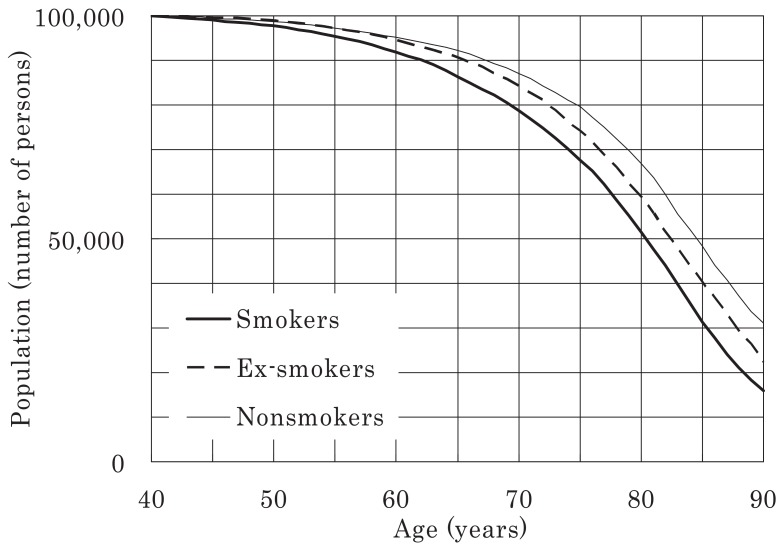
Survival curves for all males included in the study, starting from age 40 years, for a population of 100,000.

**Figure 4.**
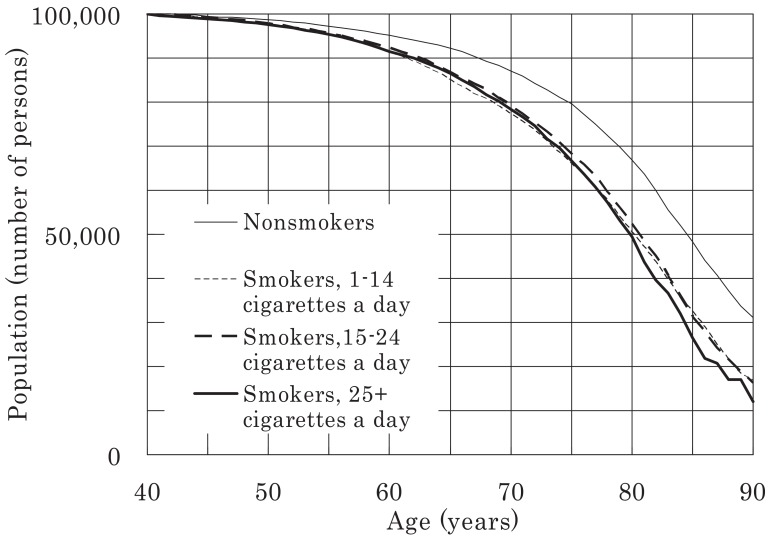
Survival curves for male smokers, starting from age 40 years, for a population of 100,000.

**Figure 5.**
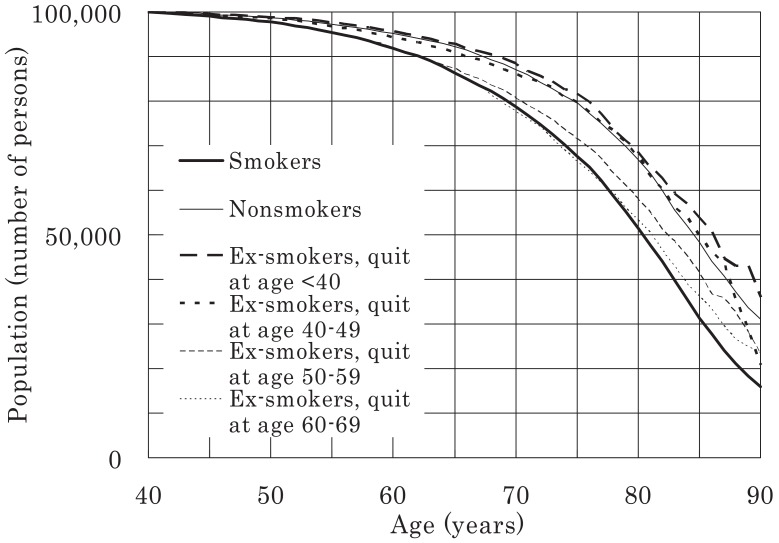
Survival curves for male ex-smokers, starting from age 40 years, for a population of 100,000.

**Figure 6.**
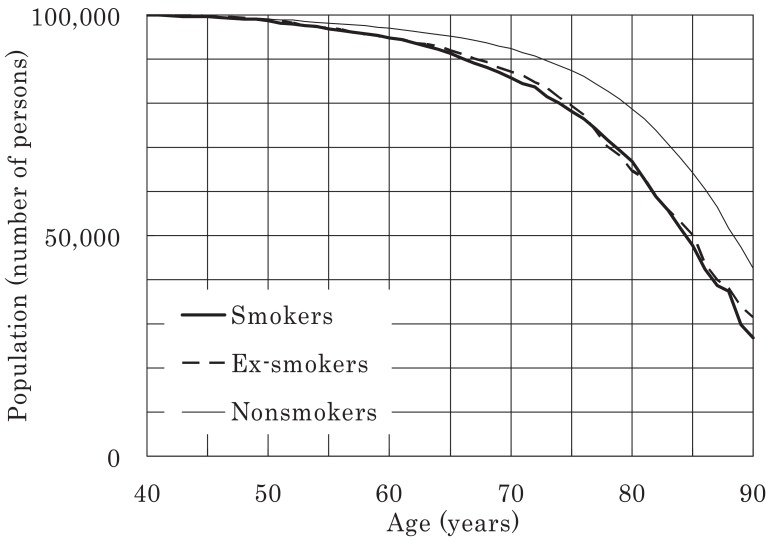
Survival curves for all females included in the study, from age 40 years, for a population of 100,000.

Survival curves (commencing at age 40 years) were plotted for all the men and women in the study for a population of 100,000 and were classified according to smoking status, as shown in Figures 3-6. Figure 3 shows that 21% of male smokers would die by 70 years of age, whereas only 13% of male never-smokers would die by the same age. For women, the corresponding proportions for smokers and never-smokers were 14% and 8%, respectively (Figure 6). For male ex-smokers, the survival curve was between those of the smokers and never-smokers (Figure 3), whereas in women, the survival curves of ex-smokers and smokers were similar (Figure 6). Amongst male smokers, the survival curve of light smokers was similar to that of heavy smokers rather than that of never-smokers (Figure 4). The survival curve of male ex-smokers who quit before age 40 was better than that of never-smokers (Figure 5). Male ex-smokers who quit at age 40-49 showed similar survival to that of never-smokers. Those quitting at ages 50-59 and 60-69 showed intermediate survival, i.e., between those of smokers and never-smokers. Survival curves for female ex-smokers quitting at various ages were between those of smokers and never-smokers, most of whom were aged < 85 years.

The age by which half of the study population had died is shown in Table 3. In males, this age was 4.2 years lower in smokers than in never-smokers, whereas the difference was 4.0 years for female smokers compared with female never-smokers. For male ex-smokers, the age at death was 2.2 years younger than that for never-smokers and the age at death for women ex-smokers compared with that for never-smokers was 3.4 years lower.

## DISCUSSION

In this study, life expectancy for male smokers aged 40 years was 3.9 years shorter than that for male never-smokers and 1.6 years shorter than that for ex-smokers. For women, the corresponding differences were 3.6 and 3.3 years. The respective life expectancies of male ex-smokers who quit smoking before ages 40, 50, 60, and 70 years were 4.8, 3.7, 1.6, and 0.5 years longer than those of smokers. Although smoking cessation at any age led to a certain recovery of life expectancy, the earlier the cessation, the larger was the recovery, and never smoking is the best way to live out our natural lives.

This study was based on data from cohort studies, but a current life table was constructed from the age-specific death rates calculated from the cross-sectional summation of observed person-years and the number of deaths at each age. Cohort subjects in age ranges 40-59, 40-69, and 40-79 years were followed up during the 1990s over approximately 10 years. We compared the life expectancy in this study with the life table to Japan in 1995, which was constructed at around the mid-point of the follow-up period of our examined cohorts.^17^ Life expectancy at age 40 years for the entire population in this study was 40.2 years for men and 46.3 years for women, whereas in the 1995 life table for Japan, the life expectancies for men and women were 37.9 and 43.9 years respectively.^17^ These figures were not directly comparable because the methods used to calculate them were different. The difference between smoking status should be considered.

The difference in median survival between smokers and never-smokers was approximately 4 years in both sexes (Tables 2 and 3). This was in contrast to the difference in the median survival (7.5 years) of smokers and nonsmokers in a 40-year study of male British physicians.^2^ In this study, the difference was 5 years in the first 20 years of observation (1951-1971) and 8 years in the second half of the study period (1971-1991). In a subsequent study of male British physicians who were born in 1900-1930 and followed up over 50 years, the difference in the median survival between smokers and nonsmokers increased to 10 years.^3^ The authors suggested that the difference between the observed median survivals calculated for the time periods 1951-1971 and 1971-1991 was because most of the deaths in nonsmokers occurred in the second half of the study.^3^ The greater difference reported in 2004 was based on direct longitudinal observation over half a century.^3^ This was because nonsmokers actually survived longer than their predicted life expectancy calculated from cross-sectional data. The median survival difference of 4 years observed in the present study, which was based on an approximately 1-decade follow-up period, is similar to that seen in the 1951-1971 part of the study on British physicians.^2^ With a longer follow-up period and decreasing prevalence of smokers, we would have observed a greater difference in the median survival time.

In our study, the difference in the survival curves for light and heavy male smokers was small. This finding is consistent with the results of Hirayama’s cohort study, which found that the relative risks of all causes of death were similar in smokers who consumed 1-9, 10-19, and 20+ cigarettes a day (relative risk: 1.35, 1.25, and 1.29, respectively); however, most individual diseases associated with smoking, such as lung cancer, showed dose-dependency.^18^

When considering the survival of ex-smokers, several points should be kept in mind. The reasons for cessation of smoking are many and varied; some smokers may quit because of illness, whereas others quit not because they are ill, but in order to avoid the known, long-term effects of smoking. In the former, the mortality rate of ex-smokers may be raised just after quitting, while in the latter, the mortality rate may be decreased for some time after quitting smoking.

Male ex-smokers who quit smoking before the age of 40 years (mean age 31 years) demonstrated an improved survival over never-smokers. These ex-smokers might have been health conscious and consequently healthier, in general, than never-smokers because they quit smoking when they were young, i.e., before the early 1990s, when the adverse effects of smoking were less well known in Japan. Some of the smokers in this category may have quit because of illness, but their proportion appeared to be small because of the longer survival of ex-smokers who quit early. In the longitudinal study of British physicians, a similar result was demonstrated for ex-smokers who quit before the age of 35.^3^ In our study, the survival of ex-smokers who quit smoking at 40-49 years of age was similar to that of never-smokers. In contrast, British physicians who quit smoking at ages 35-44 and 45-55 demonstrated reduced survival.^3^

An explanation for this difference may be that never-smokers in our Japanese study might have been less healthy, because smoking was very common in Japanese men, and the smoking rate was very high (approximately 80%) in the 1970s and 1980s.^19^ Consequently, Japanese never-smokers might have had health problems that compelled them to avoid smoking. In British physicians, although the prevalences of smokers, ex-smokers, and never-smokers were 62%, 13%, and 25%, respectively in 1951, the corresponding figures in the 1990-91 survey were 18%, 60%, and 22%, respectively.^2^ In addition, among young physicians aged 20-24 and 25-29 years in 1951, the prevalence of never-smokers was 43% and 30%, respectively.^20^ Thus, British physicians quit smoking or chose to never smoke a very long time ago.

In a previous Japanese study (NIPPON DATA 80), the difference in the life expectancies of smokers and never-smokers was 3.5 years in males and 2.2 years in females.^10^ These life expectancies are 0.4 and 1.4 years shorter than the comparable figures in our study. The baseline survey for the previous Japanese study was conducted in 1980, approximately 10 years earlier than our own, at a time when smoking was more common in Japan.^19^ Therefore, the nonsmoking group was thought to include people with conditions that compelled them to avoid smoking, and the difference in their life expectancy and that of smokers might be considered small. In addition, passive smoking, being more common in that era, could have contributed to the small survival difference by increasing the death rate of never-smokers.

There were some limitations in our study. Misclassification of smokers and never-smokers may have occurred. For example, those classified as smokers at the time of baseline survey, who subsequently quit smoking, could have contributed to a lower mortality rate in the smoker group because of improved health. This misclassification may be largely because of contemporaneous tobacco-free promotions. Such misclassification may also have occurred in our study. To reduce this possibility, it would be useful to collect data regarding changed smoking status during follow up. In women, the small number of observations in smokers and ex-smokers may have decreased the reliability of their results although the survival of heavy smokers was shorter than that of light smokers, and the survival of ex-smokers was between those of never-smokers and smokers.

Reduced life expectancy due to smoking has been shown in previous studies. In the United States, the life expectancy of smokers of both sexes was reported to be approximately 7 years less than that of nonsmokers, as determined from data sets including smoking status just prior to death.^4^ In Australia, in the mid-1980s, the difference in the life expectancies of 15-year-old males who had never smoked and those who were heavy smokers was estimated as 5.6 years. However, this estimate was based on a projection using age-specific mortality and an etiological fraction for smoking determined by the indirect method.^5^ Based on population studies in Copenhagen, the reduction in the life expectancy of heavy smokers was 9.2 years in men and 9.4 years in women; this difference is large compared with other studies and may be because data regarding changed smoking status was repeatedly collected during follow up.^6^ In a Danish National Cohort Study, the life expectancy at age 20 was 7 years less for heavy smokers than for subjects who had never smoked, and that at age 65 years was 5 years less in both men and women smokers. This was determined by estimating smoking-attributable mortality rates and using them for constructing a life table.^7^ In the Chicago Heart Association Detection Project in Industry Study, the life expectancies of male current smokers were 5.3 and 5.7 years shorter than those of never-smokers in the 2 groups with lower cholesterol levels; the life expectancies were estimated using absolute risk and absolute excess risk.^8^ In the Framingham Heart Study, the difference in the life expectancy at age 50 between subjects who had never smoked and those classified as always smokers was reported as 8.66 years in men and 7.59 years in women; in this study, the smoking status was determined in biennial exams during follow up.^9^

In conclusion, the life expectancy of the population included in Japanese large-scale cohort studies was reduced by slightly less than 4 years in smokers as compared with never-smokers in both men and women. Smoking cessation at any age led to a certain recovery of life expectancy, and the earlier the cessation, the larger was the recovery. Further, never smoking is the best way to live out our natural lives. The 4-year reduction in life expectancy may be an underestimation because in this study, the smoking status was determined only at the time of the baseline surveys for the cohort studies. In addition, in Japan around 1990, the never-smoker subset may have included people with conditions that compelled them to avoid smoking.
