# Ozasa K, et al. Reduced life expectancy due to smoking in large-scale cohort studies in Japan. *J Epidemiol *2008; 18(3): 111-118

**DOI:** 10.2188/jea.JE2008E1

**Published:** 2008-10-01

**Authors:** Kotaro Ozasa

On page 111, the second line of the left column, the values of ’42.4, 42.1 and 46.1 years’ in women should be ’42.5, 42.8 and 46.1 years’

On page 115, the forth line of the left column, the value of ’43.2’ should be ’42.4’

On page 115, the sixth line of the left column, the value of ’42.4’ should be ’42.5’

On page 115, the lowest line of the left column, the value of ’43.1’ should be ’42.8’

On page 115, the first line of the right column, the value of ’46.8’ should be ’46.1’

The editorial board regrets these errors.

